# Noninvasive positive pressure ventilation for acute respiratory failure in children: a concise review

**DOI:** 10.1186/2110-5820-1-15

**Published:** 2011-05-26

**Authors:** Abolfazl Najaf-Zadeh, Francis Leclerc

**Affiliations:** 1 Univ Lille Nord de France, UDSL, EA 2694, F-59000 Lille, France; 2 Pediatric Emergency and Infectious Diseases Unit, Roger-Salengro Hospital, Rue E Laine, CHU Lille, F-59037 Lille, France; 3 Paediatric Intensive Care Unit, Jeanne-de-Flandre Hospital, CHU Lille, Avenue E Avinée, F-59037 Lille, France

## Abstract

Noninvasive positive pressure ventilation (NPPV) refers to the delivery of mechanical respiratory support without the use of endotracheal intubation (ETI). The present review focused on the effectiveness of NPPV in children > 1 month of age with acute respiratory failure (ARF) due to different conditions. ARF is the most common cause of cardiac arrest in children. Therefore, prompt recognition and treatment of pediatric patients with pending respiratory failure can be lifesaving. Mechanical respiratory support is a critical intervention in many cases of ARF. In recent years, NPPV has been proposed as a valuable alternative to invasive mechanical ventilation (IMV) in this acute setting. Recent physiological studies have demonstrated beneficial effects of NPPV in children with ARF. Several pediatric clinical studies, the majority of which were noncontrolled or case series and of small size, have suggested the effectiveness of NPPV in the treatment of ARF due to acute airway (upper or lower) obstruction or certain primary parenchymal lung disease, and in specific circumstances, such as postoperative or postextubation ARF, immunocompromised patients with ARF, or as a means to facilitate extubation. NPPV was well tolerated with rare major complications and was associated with improved gas exchange, decreased work of breathing, and ETI avoidance in 22-100% of patients. High FiO_2 _needs or high PaCO_2 _level on admission or within the first hours after starting NPPV appeared to be the best independent predictive factors for the NPPV failure in children with ARF. However, many important issues, such as the identification of the patient, the right time for NPPV application, and the appropriate setting, are still lacking. Further randomized, controlled trials that address these issues in children with ARF are recommended.

## Introduction

**Figure 1:**
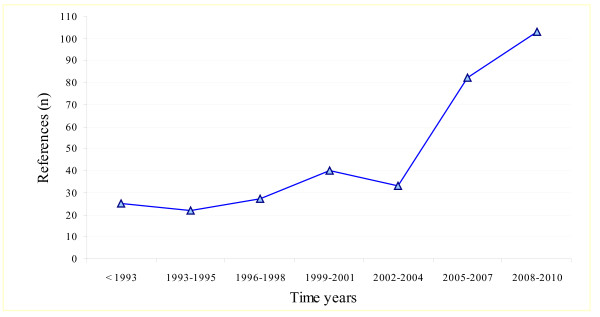
**Time course of published references on noninvasive mechanical ventilation in children aged 1 month to 18 years**.

Breathing difficulties are common symptoms in children and common reason for visits to the emergency department [1]. In United Kingdom, respiratory illnesses (both acute and chronic) accounted for 20% of weekly general practitioner consultations, 15% of hospital admissions, and 8% of deaths in childhood in 2001 [2]. Although the great majority of cases are benign and self-limited, requiring no intervention, some patients will require a higher level of respiratory support. Invasive mechanical ventilation (IMV) is a critical intervention in many cases of acute respiratory failure (ARF), but there are definite risks associated with endotracheal intubation (ETI) [3]. By providing respiratory support without ETI, non-invasive positive pressure ventilation (NPPV) may be, in appropriately selected patients, an extremely valuable alternative to IMV. It is generally much safer than IMV and has been shown to decrease resource utilization and to avoid the myriad of complications associated with ETI, including upper airway trauma, laryngeal swelling, postextubation vocal cord dysfunction, and nosocomial infections [3]. NPPV usually refers to continuous positive airway pressure (CPAP) or bilevel respiratory support, including expiratory positive airway pressure (EPAP) and inspiratory positive airway pressure (IPAP), i.e., biphasic positive airway pressure (BIPAP) and bilevel positive airway pressure (BiPAP), delivered through nasal prongs, facemasks, or helmets. Although there is high-level evidence in the literature to support the use of NPPV for the treatment of ARF due to different causes, such as exacerbation of chronic obstructive pulmonary disease [4] and acute cardiogenic pulmonary edema [5] in adults, there are few reports about its use in this acute setting in children. So far, case series constitute the vast majority of the available knowledge in this age group. However, there is an increasing interest in the use of NPPV as a therapeutic tool for children with respiratory distress that is clear from the increasing number of published studies over time (Figure 1); a research of studies on the use of NPPV in children > 1 month of age, published before December 30, 2010 (database: MEDLINE via PubMed; keywords: noninvasive ventilation, non-invasive ventilation, noninvasive positive pressure ventilation, non-invasive positive pressure ventilation, bipap, continuous positive airway pressure; age limits: children from 1 month to 18 years old) identified 332 relevant articles, of which 48% were published during the past 5 years. This concise review is designed to focus on the effectiveness of NPPV in children > 1 month of age with ARF (excluding patients with neurologic or chronic lung disease).

## Acute respiratory failure in children

The frequency of ARF is higher in infants and young children than in adults. This difference can be explained by defining anatomic compartments and their developmental differences in pediatric patients that influence susceptibility to ARF [6]. In addition, respiratory failure often precedes cardiopulmonary arrest in children, unlike in adults where primary cardiac disease often is responsible. Therefore, prompt recognition and treatment of pediatric patients with pending respiratory failure can be lifesaving [6].

Respiratory failure is a syndrome in which the respiratory system fails in one or both of its gas exchange functions: oxygenation and carbon dioxide elimination. In general, patients with respiratory failure may be classified into two groups, depending on the component of the respiratory system that is involved: hypoxemic respiratory failure and hypercapnic respiratory failure [7].

### Hypoxemic respiratory failure (known as type I)

Hypoxemic respiratory failure (type I) can be associated with virtually all acute diseases of the lung, such as status asthmaticus, bronchiolitis, pneumonia, and pulmonary edema, which interfere with the normal function of the lung and airway. The predominant mechanism in type I failure is uneven or mismatched ventilation and perfusion (intrapulmonary shunt) in regional lung units. This is the most common form of respiratory failure, characterized by a PaO_2 _< 60 mmHg with a normal or low PaCO_2_. The primary treatment of type I respiratory failure in children is to administer supplemental oxygen at a level sufficient to increase the arterial oxygen saturation (SaO_2_) to greater than 94%. In situations when a fraction of oxygen in inspired gas (FiO_2_) of greater than 0.5 is necessary to achieve this goal, this often is referred to as "acute hypoxemic respiratory failure" [7]. In this setting, NPPV may be considered.

### Hypercapnic respiratory failure (known as type II)

Hypercapnic respiratory failure (type II) is a consequence of ventilatory failure and can occur in conditions that affect the respiratory pump, such as depressed neural ventilatory drive, acute or chronic upper airway obstruction, neuromuscular weakness, marked obesity, and rib-cage abnormalities. Alveolar hypoventilation is characterized by a PaCO_2 _> 50 mmHg [7]. The onset of type II failure may be insidious and may develop when respiratory muscle fatigue complicates preexisting disorders, such as pneumonia or status asthmaticus, which present initially with hypoxemia without hypoventilation. Aministration of oxygen alone is not an appropriate treatment for hypercapnic respiratory failure and can result in the patient retaining even more carbon dioxide, especially in situations where the child has adapted to chronic hypercapnia and is relatively dependent on oxygen-sensitive peripheral chemoreceptors to maintain ventilatory drive. In addition to supplemental oxygen, therapies to reduce the load on the respiratory muscles and increase the level of alveolar ventilation should be instituted in children with type II respiratory failure.

## When to use NPPV for acute respiratory failure?

When the cause of ARF is reversible, medical treatment works to maximize lung function and reverse the precipitating cause, whereas the goal of ventilatory support is to "gain time" by unloading respiratory muscles, increasing ventilation, and thus reducing dyspnea and respiratory rate and improving gas exchange. Two recent physiological studies have demonstrated these beneficial effects of NPPV in children with ARF [8,9]. NPPV is increasingly used for treatment of ARF in children. Tables 1 and 2 summarize the studies reporting the effectiveness of NPPV in children with ARF of various etiologies [8,10-36]. However, the determinants of success of NPPV relate more prominently to the primary diagnosis as discussed below.

**Table 1:** NPPV in pediatric ARF from different causes

Study	Cause of ARF (n)	Location, Patients (n)	Age (yr)	NPPV type, Interface	Avoided ETI (%)	Other reported outcomes
**ARF due to acute airway obstruction**						

**Beers et al. **[10] retrospective	Status asthmaticus	ED, 73	2-17^a^	BiPAP Nasal mask	97	Improved RR, SaO_2_ Avoided PICU admission: 22% Major complication: 0%
**Carroll et al. **[11] retrospective	Status asthmaticus	PICU, 5	9.6^b^	BiPAP Nasal mask	100	Improved RR, MPIS Major complication: 0%
**Needleman et al. **[12] prospective, physiological	Status asthmaticus	PICU, 15	8-21^a^	BiPAP Nasal mask	-	Improved RR, thoracoabdominal synchrony, fractional inspired time: 80%
**Akingbola et al. **[13] case reports	Status asthmaticus	PICU, 3	9-15^a^	BIPAP Nasal mask	100	Improved RR, PaCO_2_, pH Major complication: 0%
**Till et al. **[14] prospective, randomized, crossover	Acute lower airway obstruction	PICU, 16	4 (0.2-14)^a,c^	BiPAP Nasal or facial mask	-	Improved RR, CAS, O_2 _requirement Major complication: 0%
**Yanez et al. **[15] multicentric, prospective, randomized, controlled (NPPV subgroup)	Bronchiolitis- pneumonia (18), asthma (4), pneumonia (3)	PICU, 25	1.3 (0.1-13)^a,c^	BIPAP, BiPAP Facial mask	72	Improved RR, HR, PaO_2_/FiO_2 _at 1 hr Major complication: 4% (interstitial emphysema)
**Thia et al. **[16]^d^ prospective, randomized, crossover	Bronchiolitis	PICU, 29	0.2 (0.1-0.4)^c,e^	CPAP Nasal prongs	-	Improved PaCO_2_ Major complication: 0%
**Cambonie et al. **[17]^d^ prospective, physiological	Bronchiolitis	PICU, 12	0.1^b^	CPAP Nasal mask	100	Improved HR, P_tc_CO_2 _, O_2_ requirement, respiratory distress score, MABP at 1 hr Major complication: 0%
**Javouhey et al. **[18]^d ^retrospective (NPPV subgroup)	Bronchiolitis	PICU, 15	0.1^c^	BiPAP, CPAP Nasal mask	67	Major complication: 7% (bacterial pulmonary coinfections)
**Larrar et al. **[19]^d^ prospective, noncontrolled (NPPV subgroup)	Bronchiolitis	PICU, 53	0.1 (0.01-1)^a,b^	CPAP Nasal prongs	75	Improved RR, PaCO_2 _at 2 hrs Death: 0% Major complication: 0%
**Campion et al. **[20]^d,f^ prospective, noncontrolled (NPPV subgroup)	Bronchiolitis-pneumonia	PICU, 69	0.1 (0.03-1)^a,c^	BIPAP, CPAP Nasal prongs, facial mask	83	Improved PaCO_2_, pH at 2 hrs Death: 0% Major complication: 0%
**Essouri et al. **[21] prospective, randomized, controlled	Laryngomalacia (5), tracheomalacia (3), others (2)	PICU, 10	0.8 (0.2-1.5)^a,c^	BiPAP, CPAP Nasal mask	-	Improved RR, respiratory effort in both types of NPPV Patient-ventilator asynchrony with BiPAP
**Padman et al. **[22]^f^ prospective, noncontrolled (upper airway obstruction subgroup)	Inspiratory stridor	PICU, 3	13^b^	BiPAP Nasal mask	100	Improved RR, HR, gas exchange, serum HCO_3_, dyspnea score at 72 hrs Major complication: 0%

**ARF due to parenchymal lung disease**						

**Munoz-Bonet et al. **[23]]^f^ prospective, noncontrolled (pneumonia subgroup)	Pneumonia	PICU, 13	0.2-15.8^a^	BIPAP Facial mask	100	Improved RR, HR, PaCO_2_, SaO_2_, pH, clinical score within the first 6 hrs Death: 0% Major complication: 0%
**Bernet et al. **[24]^d^ prospective, noncontrolled (pneumonia subgroup)	Pneumonia	PICU, 14	2.4 (0.01-18)^g^	BIPAP, CPAP Nasal or facial mask	50	Improved RR, HR, PaCO_2_, serum HCO_3 _within the first 8 hrs Death: 0%
**Fortenberry et al. **[25]^f^ retrospective, (pneumonia subgroup)	Pneumonia	PICU, 21	0.7-17^a^	BiPAP Nasal mask	90	Improved RR, PaCO_2_, PaO_2_, pH, SaO_2_, PaO_2_/FiO_2 _at 1 hr Death: 5% Major complication: 0%
**Joshi et al. **[26] retrospective (primary parenchymal lung disease subgroup)	Pneumonia, ARDS	PICU, 29	13^c^	BiPAP Facial mask	62	Improved RR, PaCO_2_, O_2 _requirement Major complication: 0%
**Essouri et al. **[27] retrospective (primary parenchymal lung disease subgroup)	CAP (23), ARDS (9), ACS (9)	PICU, 41	8 (0.2-16)^a,b^	BIPAP Nasal or facial mask	87 (CAP) 22 (ARDS) 100 (ACS)	Improved RR, PaCO_2 _at 2 hr Death: 4% (CAP), 22% (ARDS), 0% (ACS) Major complication: 0%
**Padman et al. **[22]^f^ prospective, noncontrolled (primary parenchymal lung disease subgroup)	Pneumonia (13), ACS (5 episodes)	PICU, 17	10.6^b^	BiPAP Nasal mask	85 (CAP) 80 (ACS)	Improved RR, HR, gas exchange, serum HCO_3_, dyspnea score at 72 hrs Major complication: 0%
**Padman et al. **[28] retrospective	ACS (25 episodes)	Inpatient ward, 9	11.8 (4-20)^a,b^	BiPAP Nasal mask	100	Improved RR, HR, SaO_2_, O_2 _requirement Avoided PICU admission: 44%

ACS, acute chest syndrome; ARDS, acute respiratory distress syndrome; ARF, acute respiratory failure; BiPAP, bilevel positive airway pressure; BIPAP, biphasic positive airway pressure; CAS, clinical asthma score; CAP, community-acquired pneumonia; CPAP, continuous positive airway pressure; ED, emergency department; ETI, endotracheal intubation; FiO_2_, fraction of oxygen in inspired gas; HR, heart rate; MABP, mean arterial blood pressure; MPIS, modified pulmonary index score; NPPV, noninvasive positive pressure ventilation; PICU, pediatric intensive care unit; PaCO_2_, arterial partial pressure of carbon dioxide; PaO_2_, arterial partial pressure of oxygen; P_tc_CO_2_, transcutaneous PCO_2_; RR, respiratory rate; SaO_2_, arterial oxygen saturation.

^a^Range.

^b^Mean.

^c^Median.

^d^Neonatal cases also were included in the study.

^e^Interquartile range.

^f^Certain patients included in the study had underlying neurologic or chronic lung disease.

^g^The numbers represent the median (range) age of the patients (n = 42) with ARF of various causes included in the study.

**Table 2:** NPPV in specific circumstances

Study	Cause of ARF (n)	Location, Patients (n)	Age (yr)	NPPV type, Interface	Avoided ETI (%)	Other reported outcomes
**NPPV in postoperative ARF**						

**Stucki et al. **[8]^a^ prospective, crossover (cardiac surgery)	Interstitial pulmonary oedema	PICU, 6	0.4 (0.04-0.6)^b,c^	BIPAP Nasal mask	100	Improved RR, PTPes, dPes, dyspnea score Death: 0%
**Bernet et al. **[24]^a^ prospective, noncontrolled (cardiac surgery subgroup)	ND	PICU, 11	2.4 (0.01- 18)^d^	BIPAP, CPAP Nasal or facial mask	64	Improved RR, HR, PaCO_2_, pH, serum HCO_3 _within the first 8 hrs Death: 0%
**Joshi et al. **[26] retrospective (postoperative subgroup)	Atelectasis	PICU, 16	12^b^	BiPAP Facial mask	94	Improved RR, PaCO_2_, O_2 _requirement, SaO_2_ Major complication: 0%
**Essouri et al. **[27]^a^ retrospective (postextubation subgroup)^e^	ND	PICU, 61	3.2 (0.04- 15)^c,f^	BIPAP Nasal or facial mask	67	Improved RR, PaCO_2 _at 2 hrs Death: 11% Major complication: 0%
**Kovacikova et al. **[29] case reports (cardiac surgery)	Bilateral diaphragm paralysis	PICU, 2	0.9-3.5^c^	BIPAP Facial mask, Nasopharyngeal tube	100	Improved RR, gas exchange Major complication: 100% (respiratory tract infection)
**Chin et al. **[30] retrospective (liver transplantation)	Atelectasis, hypercapnia +/-hypoxemia, pleural effusion, pneumonia	PICU, 15	0.2-14^c^	BiPAP Nasal or facial mask	87	Improved PaCO_2_, SaO_2_, atelectasis Death: 13%

**NPPV for facilitation of ventilation weaning/rescue of failed extubation (not postoperatively)**						

**Lum et al. **[31]^a^ prospective, noncontrolled (prior IMV subgroup)	Post-extubation failure (51), weaning facilitation (98)	PICU, 149	0.5 (0.1- 2)^b,g^	BiPAP Nasal or facial mask	75 (failure group), 86 (weaning group)	Improved RR, HR, FiO_2 _within the first 24 hrs Death: 5% Major complication: 11% (pneumonia)
**Mayordomo-Colunga et al. **[32]^a,h^ prospective, noncontrolled	Post-extubation failure (20), weaning facilitation (21)	PICU, 36 (41 episodes)	1.7 (0.04- 17)^b,c^	BiPAP, CPAP Nasal or facial mask, helmet	50 (failure group), 81 (weaning group)	Death: 5% Major complication: 5% (hypercapnia), 12% (upper airway obstruction), 7% (apnea), 10% (other)

**NPPV in immunocompromised patients**						

**Munoz-Bonet et al. **[23] prospective, noncontrolled (immunocompromised subgroup)	Pnemonia (3), ARDS (5)	PICU, 8	1.5-13.8^c^	BIPAP Facial mask	100 (pneumonia), 40 (ARDS)	Improved RR, HR, PaCO_2_, SaO_2_, pH, clinical score within the first 6 hrs Death: 0% Major complication: 0%
**Essouri et al. **[27] retrospective (immunocompromised subgroup)	ND	PICU, 12	8 (3-16)^c,f^	BIPAP Nasal or facial mask	92	Improved RR, PaCO_2 _at 2 hrs Death: 8%, Major complication: 0%
**Schiller et al. **[33] retrospective	Pneumonia (5), ARDS (10), pulmonary mass (1)	PICU, 14 (16 episodes)	13.3^f^	BiPAP Facial mask	80	Improved RR, PaO_2 _at 1 hr Death: 20% Major complication: 0%
**Piastra et al. **[34] prospective, noncontrolled	ARDS	PICU, 23	10.2^f^	BIPAP Facial mask, Helmet	54	Improved gas exchange at 1 hr (82%), sustained (74%) Death: 35% Major complication: 0%
**Desprez et al. **[35] case reports	Pneumonia (1), ARDS (1)	PICU, 2	13-14^c^	BIPAP Facial mask	100	Death: 0% Major complication: 50% (upper and lower digestive hemorrhage)
**Pancera et al. **[36] retrospective (NPPV subgroup)	ND	PICU, 120	9^i^	BIPAP Nasal mask	74	Death: 22.5%

ARDS, acute respiratory distress syndrome; ARF, acute respiratory failure; BiPAP, bilevel positive airway pressure; BIPAP, biphasic positive airway pressure; CPAP, continuous positive airway pressure; dPes, oesophageal inspiratory pressure swing; ETI, endotracheal intubation; HR, heart rate; IMV, invasive mechanical ventilation; ND, not determined; NPPV, noninvasive positive pressure ventilation; PaCO_2_, arterial partial pressure of carbon dioxide; PaO_2_, arterial partial pressure of oxygen; PICU, pediatric intensive care unit; PTPes, oesophageal pressure-time product; RR, respiratory rate; SaO_2_, arterial oxygen saturation.

^a^Neonatal cases also were included in the study.

^b^Median.

^c^Range.

^d^Numbers represent the median (range) age of the patients (n = 42) with ARF of various causes included in the study.

^e^32 patients were intubated for liver transplantation, 11 for other abdominal surgery, and 18 for respiratory distress.

^f^Mean.

^g^Interquartile range.

^h^Certain patients included in the study had underlying neurologic disease.

^i^Number represent the mean age of the patients (n = 239) included in the study, of which 120 had NPPV.

## NPPV in pediatric ARF from primary respiratory disease

### Acute lower airway obstruction

Lower airway disease is a common cause of ARF. Asthma accounts for the largest percentage of this group, but infections, such as viral bronchiolitis, also are common and predominantly impact the small airways. Physicians caring for acutely ill children are regularly faced with this condition. Both non-invasive and invasive ventilation may be options when medical treatment fails to prevent respiratory failure. ETI and positive pressure ventilation in children with lower airway obstruction may increase bronchoconstriction, increase the risk of airway leakage, and has disadvantageous effects on circulation and cardiac output. Therefore, ETI should be avoided unless respiratory failure is imminent despite adequate institution of all available treatment measures. NPPV can be an attractive alternative to IMV for these patients. Clinical trials in children with acute lower respiratory airway obstruction have suggested that NPPV may improve symptoms and ventilation without significant adverse events and reduce the need for IMV [10-20]. NPPV theoretically improves the respiratory status of patients with lower respiratory airway obstruction by several mechanisms [37]. During acute bronchospastic episodes, patients have an increase in airway resistance and expiratory time constant. The combination of prolonged expiratory time constant and premature closure of inflamed airways during exhalation results in dynamic hyperinflation, which causes increased positive pressure in the alveoli at end-expiration (auto-PEEP). Because the alveolar pressure must be reduced to subatmospheric levels to initiate the next breath, this auto-PEEP increases the inspiratory load and induces respiratory muscle fatigue. The EPAP delivered by NPPV may help to decrease dynamic hyperinflation by maintaining small airway patency and may reduce the patient's work of breathing by decreasing the drop in alveolar pressure needed to initiate a breath. In addition, inspiratory support, i.e., IPAP delivered by NPPV, helps to support fatigued respiratory muscles, thereby improving dyspnea and gas exchange. Needleman et al., in a physiological study, found that the NPPV use in children with status asthmaticus was associated with a decrease in respiratory rate and fractional inspired time and an improvement of thoracoabdominal synchrony in 80% of patients [12]. A few clinical studies of small size (3-73 patients) reported the use of NPPV for treatment of status asthmaticus in children (Table 1) [10,11,13,14]. NPPV was well tolerated with no major complications and was associated with an improvement of gas exchange and respiratory effort (Table 1).

Viral bronchiolitis, mainly due to respiratory syncytial virus, represents the largest cohort of children treated with NPPV [15-20]. Use of NPPV in infant with severe bronchiolitis was associated with improved respiratory rate [15,19] and PaCO_2 _[16,19,20], decreased work of breathing [17], and ETI avoidance in 67-100% of patients (Table 1) [17,18].

### Acute upper airway obstruction

In children, dynamic upper airway obstruction can present as an acute life-threatening condition and leads to severe alveolar hypoventilation. In 2006, a survey of French PICU group found that 67% of pediatric intensivists applied frequently or systematically NPPV in the management of dynamic upper airway obstruction in children [38]. However, there is a paucity of literature on the use of NPPV in the acute setting of upper airway obstruction in children. NPPV was associated with a significant decrease in respiratory effort [21] and a sustained improvement in gas exchange [22] in children with dynamic upper airway obstruction (Table 1).

### Parenchymal lung disease

The main goals of NPPV in patients with parenchymal lung disease, such as pneumonia, acute lung injury (ALI), and acute respiratory distress syndrome (ARDS), are to improve oxygenation, to unload the respiratory muscles, and to relieve dyspnea. The first goal can usually be achieved by using EPAP to recruit and stabilize previously collapsed lung tissue [39]. Unloading of the respiratory muscles during NPPV with IPAP has been reported by L'Her et al. in adult patients with ALI [39]. The authors concluded that adding IPAP to EPAP may be indispensable in patients with ALI treated with NPPV [39]. Indeed, IPAP allows a better respiratory system muscle unloading, alveolar recruitment, oxygenation, and CO_2 _washout improvement.

Although NPPV seems disappointing in ARF owing to pneumonia in adult patients, with failure rates of up to 66% [40], several noncontrolled trials have suggested that NPPV could improve symptoms and ventilation without significant adverse events and reduce the need for IMV in children with ARF due to pneumonia [22-27]. Use of NPPV in this acute setting in children was associated with reduction in ETI rates ranging from 50-100% (Table 1) [23,24].

The most challenging application of NPPV may be in patients with ARDS. Studies of NPPV for the treatment of ARDS in adult population have reported failure rates of 50-80% [40]. A meta-analysis of the topic in adult population concluded that NPPV was unlikely to have any significant benefit [41]. In children, the use of NPPV for the treatment of ARDS was associated with a failure rate of 78%, and 22% of them died (Table 1) [27]. Therefore, NPPV use in such a patient group is rarely justified. However, if a trial of NPPV is initiated, patients should be closely monitored and promptly intubated if their conditions deteriorate, so that inordinate delays in needed interventions are avoided.

Acute chest syndrome (ACS) is one of the leading causes of death and hospitalization among patients with sickle cell disease [42]. Approximately 70% of patients (adults or children) with ACS are hypoxic [43]. Indeed, patients with sickle cell disease are prone to infarctive crises. Thoracic bone infarction (usually in the ribs) in such patients leads to pain, splinting, hypoventilation, and the clinical signs of ACS. *In situ *red blood cell sickling in the lung vasculature is possibly a consequence of hypoventilation with subsequent infarction of lung parenchyma. NPPV has been proposed as a therapeutic option for patients with ACS. By improving patient oxygenation, NPPV could prevent progression from painful crisis to ACS, and ultimately to ARDS. Three retrospective studies reported favorable outcomes in children with ACS treated with NPPV (Table 1) [22,27,28].

## NPPV in specific circumstances

### Postoperative respiratory failure

Postoperative pulmonary complications are a major cause of morbidity, mortality, prolonged hospital stay, and increased cost of care [44]. It has been reported that 5-10% of all surgical adult patients experience postoperative pulmonary complications [45]. Atelectasis, postoperative pneumonia, ARDS, and postoperative respiratory failure have all been classified as postoperative pulmonary complications. Postoperative respiratory failure is most commonly defined as the inability to be extubated 48 hours after surgery [46], although some investigators have used 5 days [47]. NPPV has been successfully used to treat postoperative respiratory failure in both pediatric and adult patients. Compared with standard treatment, NPPV used after major abdominal surgery improved hypoxemia and reduced the need for ETI in adult population [48]. NPPV application in children with postoperative respiratory failure was associated with improved respiratory effort, gas exchange, oxygen saturation, and reduced the need for ETI (Table 1) [8,24,26,27,29,30].

### Facilitation of ventilation weaning/rescue of failed extubation

The need for reintubation after failed extubation is associated with increased morbidity and high mortality [49]. NPPV has been proposed as a means of "facilitating" weaning from IMV, and as a "curative" treatment for postextubation respiratory failure. Although several studies have shown the efficacy of NPPV in weaning from IMV in adult population [50], its application for postextubation respiratory failure is not supported by randomized, controlled trials [51]. In children, two noncontrolled trials assessed the efficacy of NPPV in these settings: the application of NPPV as a means of "facilitating" ventilation weaning, and as "curative" treatment for postextubation respiratory failure was associated with success rates of 81-86% and 50-75%, respectively [31,32].

### Immunocompromised children

ARF in immunocompromised patients most often results from infections, pulmonary localization of the primary disease, or even postchemotherapy cardiogenic pulmonary edema. Treatment of such patients often requires intubation and mechanical ventilation. Avoidance of the infectious complications associated with IMV is particularly attractive in these high-risk patients, in whom this could be devastating, if not fatal. Results of randomized, controlled trials have proven the beneficial effects of NPPV in immunocompromised adult patients [52,53]. Some case series reported the use of NPPV in the treatment of respiratory failure in immunocompromised children (Table 2) [23,27,33-36]. The likelihood of NPPV success in immunocompromised children seems to be related rather to the type of pulmonary disease: the ETI avoidance rates varied from 40% for ARDS to 100% for pneumonia (Table 2).

## Are there predictive factors of NPPV failure in children with ARF?

It is not always apparent which patients will initially benefit from NPPV; some patients do not obtain adequate ventilation with NPPV. The NPPV failure rate may be fairly consistent for certain diseases, and NPPV failure eventually requires intubation. Inability to early identify patients who will fail NPPV can cause inappropriate delay of intubation, which can cause clinical deterioration and increase morbidity and mortality. Knowing the predictors of NPPV failure in patient with ARF is therefore crucial in deciding if and when to apply this ventilatory technique. Several authors have identified different predictive factors of NPPV failure in children with ARF: the results of studies are given in Table 3[20,24,26,27,31,54,55]. The best predictive factors for the NPPV failure in ARF appear to be the level of FiO_2 _and PaCO_2 _on admission or within the first hours after starting NPPV (Table 3).

**Table 3:** Predictive factors for the outcome of NPPV in children with ARF

Study	Population (n)	Age (yr)	Success rate (%)^a^	Predictors of failure
**Campion et al. **[20]^b,c^ prospective noncontrolled	Bronchiolitis (69)	0.1 (0.03-1)^d,e^	83	Apnea Higher PaCO_2 _on admission Higher PRISM score at 24 hrs
**Bernet et al. **[24]^b^ prospective noncontrolled	Pneumonia (14), bronchiolitis (4), postoperative ARF (11), other (13)	2.4 (0.01-18)^d,e^	57	FiO_2 _> 0.8 at 1 hr
**Joshi et al. **[26] retrospective (primary parenchymal lung disease subgroup)	Pneumonia or ARDS (29)	13^d^	62	Age ≤ 6 yr FiO_2 _> 0.6 within the first 24 hrs PaCO_2 _≥ 55 mmHg within the first 24 hrs
**Essouri et al. **[27]^b^ retrospective	CAP (23), ARDS (9), ACS (9), immune deficiency (12), postextubation ARF (61)	5.3 (0.04-16)^e,f^	73	ARDS High PELOD score
**Lum et al. **[31]^b^ prospective noncontrolled	Pulmonary diseases (129), postextubation ARF (149)	0.7 (0.3-2.8)^d,g^	76	Higher FiO_2 _needs at start of NPPV Higher PRISM score on admission Sepsis at start of NPPV
**Munoz-Bonet et al. **[54] prospective noncontrolled	Pneumonia (20), ARDS (10), postextubation ARF (11), other (6)	7.1 (0.1-16)^e,f^	81	MAP > 11.5 cm H_2_O FiO_2 _> 0.6
**Mayordomo-Colunga et al. **[55]^b^ prospective noncontrolled	Type I ARF (38), type II ARF (78)	0.9 (0.05-14)^d,e^	84	Lower RR decrease at 1 hr and 6 hrs Higher PRISM score at start and at 1 hr Type I ARF

ACS, acute chest syndrome; ARDS, acute respiratory distress syndrome; ARF, acute respiratory failure; CAP, community-acquired pneumonia; FiO_2_, fraction of oxygen in inspired gas; MAP, mean airway pressure; NPPV, noninvasive positive pressure ventilation; PaCO_2_, arterial partial pressure of carbon dioxide; PELOD, pediatric logistic organ dysfunction; PRISM, pediatric risk of mortality; RR, respiratory rate.

^a^NPPV was considered successful when endotracheal intubation was avoided.

^b^Neonatal cases also were included in the study.

^c^Certain patients included in the study had underlying neurologic or chronic lung disease.

^d^Median.

^e^Range.

^f^Mean.

^g^Interquartile range.

## Conclusions

During recent years, there has been an increasing interest in the use of NPPV for children with ARF. There are some promising studies supporting its use in this acute setting. NPPV was well tolerated with rare major complications and was associated with improved gas exchange, decreased work of breathing, and decreased need for ETI. Both critical care ventilators and portable ventilators have been used for NPPV. However, the vast majority of the available knowledge in this acute setting results from noncontrolled trials and case series of small size. As such, many important issues, such as the identification of the patient, the right time for NPPV application, and the appropriate setting, are still lacking. Further randomized, controlled trials addressing these issues in children with ARF are needed to define better the patients who are likely to benefit from this alternative method of respiratory support. Also, the respective place of NPPV and high flow oxygen therapy in children with ARF due to different conditions has to be determined [56].

## Competing interests

The authors declare that they have no competing interests.

## Authors' contributions

AN and FL contributed to query the literature and to draft the manuscript. They approved the final version.
